# The spatial spillover effect of ICT development level on regional CO_2_ emissions

**DOI:** 10.1038/s41598-023-34573-2

**Published:** 2023-05-11

**Authors:** Jinqi Su, Wenbo Wang, Shiyao Tang

**Affiliations:** 1 School of Management and Economics, Xi’an University of Posts and Telecommunications, Xi’an, China; 2 School of Modern Post, Xi’an University of Posts and Telecommunications, Xi’an, China

**Keywords:** Energy and society, Environmental impact

## Abstract

Information and communication technology plays an essential role in affecting CO_2_ emissions and promoting green and sustainable development. This paper empirically examines the effect of the level of urban ICT development on CO_2_ emissions using panel data for Chinese cities from 2010 to 2020. On this basis, a spatial Durbin model is used to analyze the spatial spillover effects of ICT development level affecting regional CO_2_ emissions, and its direct, indirect and overall effects are further analyzed. The conclusions of the study are as follows. (1) The CO_2_ emissions of Chinese cities are correlated by spatial distance and show high-high or low-low clustering characteristics. (2) ICT development can reduce CO_2_ emissions in the region and increase CO_2_ emissions in neighboring regions. This conclusion still holds after several robustness tests. And when the level of ICT development increases by 1 standard deviation, CO_2_ emissions are reduced by 64%. (3) The mechanism of action shows that ICT can influence regional CO_2_ emissions through its technological innovation effect and industrial upgrading effect. (4) The impact of ICT development on regional carbon emissions is characterized by significant heterogeneity depending on the city's geographical location and whether or not environmental information is publicly available.

## Introduction

Whether one believes or not in climate change per se, the damaging effects of GHG emissions are becoming self-evident. For instance, the increasing occurrence of thick smog-cloud over several major cities of the world for several months every year (e.g., in China^1,2^) and rising healthcare costs due to carcinogenic emissions, devastating droughts, and floods have become threatening challenges for the welfare and economic development of nations across the world^3,4^. At present, climate change has become a serious threat to the sustainable development of human society. China's energy consumption and CO_2_ emissions are rapidly increasing and have surpassed the United States to become the world's largest CO_2_ emitter^5^^.^ China's rapid economic and social development has resulted in an increasing demand for energy resources^6^. China currently relies heavily on high-CO_2_ fossil fuels, yet its resources and energy utilization are low. In the context of urbanization and industrialization, energy demand is getting bigger and bigger, which puts China under huge pressure to reduce CO_2_ emissions^7^. China's national leaders emphasized that "achieving CO_2_ peaking and CO_2_ neutrality is a broad and profound economic and social systemic change"^8^. In ’Chinese digital transformation and green development, the ICT industry needs to achieve its green and low-CO_2_ development and help other industries reduce CO_2_ emissions^10^^.^ ICT includes all the related infrastructure that allows people to record and send information, connecting the world^9^. With the deep integration and application of ICT in the fields of resources, energy and environment, the role of the ICT industry in achieving CO_2_-neutral goals is receiving increasing attention^10^. However, with the rapid development of digital technology, the energy demand and CO_2_ emissions of the ICT industry are increasing, which requires us to analyze the role of ICT development on CO_2_ neutrality dialectically.

In fact, with the further implementation of China's manufacturing power and network power strategies, energy consumption in the ICT industry is rapidly increasing. Data centers, network facilities, and terminal equipment such as cell phones, computers and TVs are the main sources of energy consumption in the ICT industry^11^. Scholars from different countries disagree about the CO_2_ emissions of the world's ICT industry in 2020, which in general is about 3–6% of the total global greenhouse gas emissions. To reach the " CO_2_ peaking and CO_2_ neutrality goals" as soon as possible, we must pay attention to the impact of greenhouse gas emissions in the ICT industry and should take measures to promote energy saving and emission reduction. Some of the leading companies have already made a head start. Some data service providers have already released their plans and goals, and have come up with green transformation programs^12,13^. In addition, Chinese three major carriers—Mobile, Unicom and Telecom are constantly looking for new ways to reduce the energy consumption of building 5G base stations. China's ICT companies have been slower to implement CO_2_ reduction measures than their international counterparts, so China should also pay attention to the problem of increasing CO_2_ emissions from the ICT industry.

Under the goal of "CO_2_ peaking and CO_2_ neutrality", the ICT industry's energy consumption problem cannot be ignored. The ICT industry released approximately 7 million tons of CO_2_ in 2012, less than 0.1% of total emissions^14^. This has led many scholars to believe that the ICT industry is environmentally friendly. So there is no need to study the CO_2_ emissions generated by it. However, some academic studies have found that the CO_2_ emissions generated by the ICT industry can be significant. GeSI (Global e-Sustainability Initiative, 2015) roughly predicts that by 2030, the ICT sector will generate 1.25 gigatons of CO_2_ emissions, accounting for 1.97% of global emissions^15^. Zhang Chuangguo examined the role of the ICT industry in CO_2_ emissions in different regions of China, and the results showed that the development of the ICT industry has different effects on CO_2_ emission reduction in different regions^16^. Belkhir and Elmeligi capture the global CO_2_ footprint of the ICT sector, suggesting that the ICT sector's emissions contribution will double^17^. Although there have been some detailed studies on the correlation between ICT development and regional CO_2_ emissions in China, the findings still vary widely. The possible reason for this is that the spatial spillover effect of ICT development on regional carbon emissions has been overlooked. However, most of the literature uses time series data and panel models to study the relationship between ICT development and regional carbon emissions. The time series models and panel models fail to take into account the impact of the spatial flow of production factors such as capital and labor between regions through information factors on local regional carbon emissions, thus failing to consider the spatial spillover effect of the impact of ICT development on regional carbon emissions.

This paper attempts to construct a comprehensive indicator system for the level of ICT development at the city level and to explain the mechanism of the role of ICT in influencing regional CO_2_ emissions. The method of spatial measurement is adopted to improve the reliability and scientificity of the research findings. On this basis, this paper puts forward policy recommendations for carbon reduction in cities. The possible marginal contributions of this paper are as follows. Firstly, this paper uses the spatial Durbin model to study the direct effects of urban ICT development level and regional CO_2_ emissions and conducts robustness tests based on other spatial regression models. Secondly, based on the technological innovation effect and industrial upgrading effect of ICT, the mechanism of action between the level of ICT development and regional carbon emissions is elaborated. The relevant research on its intrinsic mechanism of action is expanded. Third, the effect of the level of ICT development on regional carbon emissions is studied more deeply from the perspective of heterogeneity, such as the regional distribution of cities in China and whether environmental information is disclosed.

The arrangement of the paper is as follows: the second part is the literature review; the third part is the research hypothesis; the fourth part is the selection of indicators, the establishment of models and data sources; the fifth part is the empirical results and discussion of empirical results; the final part summarizes the research conclusions and policy recommendations.

## Literature review

Existing studies on the relationship between ICT development and regional CO_2_ emissions have concluded two contrasting views. Some scholars believe that ICT can improve the environment through its application in optimizing production processes and achieving better environmental management. Other studies have concluded that the environmental consequences of manufacturing, operating and disposing of ICT devices (such as electricity consumption, and e-waste) can hurt the environment.

### Studies related to ICT development to reduce regional CO_2_ emissions

On the one hand, scholars examine whether ICT development can influence regional CO_2_ emissions at a theoretical level. First, the necessity of the existence of emission reduction contribution of ICT in the development process was analyzed. Some scholars believe that the development of ICT in the production process of enterprises has led to the automation of the production process and improved the efficiency of the utilization of labor, capital, energy, and other factor resources^18^ and reduced the level of energy consumption^19^, thus contributing to energy conservation and emission reduction. The second is to study the quantification method of CO_2_ emission reduction. Wang et al. studied the CO_2_ emission reduction contribution of information and communication technologies and argued that only scientific measurement of CO_2_ emission reduction brought about can better manage the development of low CO_2_^20^.

On the other hand, scholars study the relationship between ICT development and regional carbon emissions at the level of empirical analysis. Liu et al. use an empirical analysis of Chinese inter-provincial panel data to show that informatization significantly reduces China's CO_2_ emissions^21^. Liu et al. further showed that for every 1% increase in the level of information technology development, China's sulfur dioxide emissions are reduced by 2.866%^22^. Ishida^23^, Lange et al.^24^, and Kumar and Manas^25^ using data from Japan, Germany, and India, found that as ICT investment increased, it improved energy use efficiency and reduced energy consumption intensity, which in turn helped to achieve CO_2_ emission reductions.

### Studies related to the increase of regional CO_2_ emissions by ICT development

ICT is a basic and pioneering industry that supports cities’ economic and social development, and the impact on CO_2_ emissions caused by the whole economic system is multifaceted. The generation, application and services of ICT can negatively impact the environment and increase CO_2_ emissions due to electricity generation. Mingay estimates that the ICT industry contributes 2% of global greenhouse gas emissions^26^. A study by Røpkea and Christensen concluded that the use of ICT has a direct impact on electricity consumption, even leading to energy consumption associated with the production of equipment and the operation of infrastructure, such as server parks and data centers^27^. Thakur and Chaurasia proposed a set of metrics to measure CO_2_ emissions in cloud computing and found that data centers and cloud servers are the main sources of CO_2_ emissions generated^27^. Shabani and Shahnazi studied the relationship between energy consumption, GDP, CO_2_ emissions and ICT in Iranian economic sectors and showed that ICT increases CO_2_ emissions in the industrial sector and decreases CO_2_ emission levels in the transportation and service sectors^28^.

In summary, the environmental effects of ICT development are still debated, and previous studies have only focused on the direct effects of the level of ICT development on regional CO_2_ emissions, but have not yet addressed the possible spatial correlation between regions. And this paper adopts a spatial econometric method to explain the mechanism from the perspectives of the technological innovation effect and industrial upgrading effect of ICT development and explores the spatial spillover effect of ICT development level on regional CO_2_ emissions.

## Research hypothesis

### ICT development levels and regional CO_2_ emissions

The ICT industry occupies a pivotal position in the national economy and has an important impact on China’s economic development. Modern economic growth theory considers science and technology the most essential element and core drivers of economic growth, so ICT, an emerging technology industry, can drive economic development^29^. Firstly, the use of ICT can directly increase productivity and contribute to economic growth. Secondly, an economy's green total factor productivity can be increased by taking advantage of the technological innovation effect brought about by ICT. Finally, the application of ICT is conducive to improving the efficiency of energy use and reducing energy consumption, which in turn helps to save energy and reduce emissions. At the same time, ICT is an important factor in enhancing the economic agglomeration of cities. The development of ICT has made it possible to link the economic activities of various regions into a single entity. That way can strengthen contacts and exchanges between cities, and intensify the spatial concentration of various factors in production in cities with greater technological innovation and economic development. The spatial clustering of factors of production affects regional carbon emissions in two ways. First, the concentration of production activities leads to an expansion of economic scale. The resulting increase in energy consumption can exacerbate pollution emissions. Secondly, economic agglomeration will increase the proportion of the tertiary industry, which has the characteristic of low pollution intensity. The knowledge spillover effect of modern service industries will lead to a synergistic agglomeration of modern manufacturing industries, which will help form industrial integration and enhance production efficiency, thereby reducing pollution emissions per unit of output. In summary, ICT can have an overall and multifaceted impact, with a definite impact effect on our regional carbon emissions.

H1: The level of ICT development can reduce regional CO_2_ emissions and has a spatial spillover effect.

### Technological innovation effects of ICT

As the core of the new generation of the information technology revolution, ICT is driving the development of digitalization, networking and intelligence, leading to a fundamental change in the innovation ecosystem. There are two main paths for ICT to influence carbon emissions through technological innovation. On the one hand, big data, cloud computing, the Internet of Things and industrial internet technologies penetrate the industry, which can optimize and upgrade the industry's pollution management strategy and energy management mode in multiple dimensions. At the same time, ICT can also be based on multi-dimensional sensors to form connections between enterprise departments and production equipment. By linking different networks for communication, information on various elements and energy closely related to the enterprise's emission activities can be dynamically collected in real-time, optimizing the enterprise's energy structure and usage efficiency, thus achieving energy saving and emission reduction. On the other hand, ICT can promote the linking of innovation subjects, innovation collaboration and knowledge sharing, resulting in ICT empowerment and the enhancement of urban innovation capabilities. Moreover, it can enhance the urban innovation environment through talent clustering and science and technology financial supply, accelerating the digital transformation and innovation of cities, which in turn catalyzes the technological innovation and emission reduction effect.

H2: ICT can achieve CO_2_ emission reductions through technological innovation effects.

### Industrial upgrading effects of ICT

**Figure 1:**
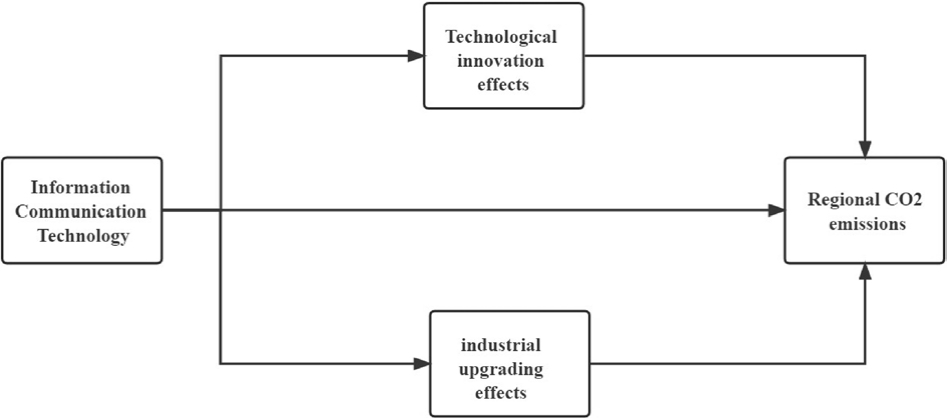
Conceptual model diagram.

The new generation of the information technology revolution, led by information and communication technology, is driving changes in production methods worldwide, changing the existing industrial supply system and technological innovation system and penetrating all aspects of business production and operation^30^. It is a proven consensus that the environmental effects of industrial upgrading are mainly achieved through externalities. The externalities of industrial upgrading mainly rely on technology externalities, i.e. knowledge and technology spillovers, which mainly include MAR externalities, Jacobs externalities and Porter externalities^31^. High-quality production factors are more likely to be concentrated from economically disadvantaged areas to economically developed areas, further strengthening the concentration of production factors in developed cities. At the same time, the "profitability" of production factors helps to optimize the spatial allocation of resources, leading to an increase in urban productivity. The increase in urban productivity, on the other hand, will help to increase the share of the tertiary sector and promote the upgrading of the industrial structure. An increase in the share of the tertiary industry will reduce pollutant emissions and lower carbon emissions in urban areas (Fig. 1).

H3: ICT can achieve CO_2_ emission reductions through industrial upgrading effects.

## Method and data

### Spatial econometric model

Previous studies on the application of spatial econometric models have mostly focused on spatial autoregressive models (SAR) containing only lags of spatial dependent variables, and spatial error models (SEM) containing only autocorrelation of spatial error terms. However, spatial effects may occur simultaneously in the transmission process due to spatial lags of variables and random shocks, which cause changes in the error term. Due to the above reasons, LeSage and Pace^32^ constructed a spatial Durbin model (SDM) and a spatial crossover model (SAC) that combined the above two spatial transmission mechanisms.

Different types of spatial econometric models do not assume the same spatial transmission mechanisms and differ in the economic meanings they represent. The SEM model assumes that spatial spillovers occur as a result of random shocks and that their spatial effects are mainly transmitted through error terms. The SAR model assumes that the explanatory variables all affect the economies of other regions through spatial interactions. The general SAC model and the SDM model consider both of these spatial transmission mechanisms, and the SDM model also considers spatial interactions. That is, the level of economic growth of a province is not only influenced by the independent variables of the province but also by the level of economic growth and the independent variables of other provinces. As can be seen, the setting and selection of the spatial econometric model are crucial.

To make the regression results more accurate, the spatial Durbin model, which can comprehensively consider the spatial correlation of economic activities of each city, will be selected for estimation in this paper. In addition, LM tests and LR tests were conducted on the spatial Durbin model, and the p-values of the tests were all significantly zero at the 1% level, indicating that the SDM model fits better. Based on this, this paper refers to the research of Bai^33^ to establish the model. The spatial Durbin model is as follows:

1 $$lnY_{it} = \, \beta_{0} + \delta WlnY_{it} + \beta_{1} lnX_{it} + \beta_{2} lnX_{Control} + \theta_{1} WlnX_{it} + \theta_{2} WlnX_{control} + \varepsilon_{it} .$$

In the formula, *Y*_*it*_ represents the value of regional CO_2_ emission level, *X*_*it*_ represents the level of development of information and communication technology (ICT), *X*_*control*_ represents a series of control variables, *W* is the spatial weight matrix, *ε*_*it*_ denotes random perturbation terms.

### Indicator selection

#### Measurement of regional CO_2_ emission levels

The regional CO_2_ emission level is the dependent variable in this paper. The existing city-scale CO_2_ emission accounting studies are mainly based on the IPCC inventory preparation guidelines and energy consumption data in the energy balance sheet, and this accounting method usually accounts for the CO_2_ emissions caused by energy consumption. In this paper, the total CO_2_ emissions from energy consumption in each region are selected as a measure of the regional CO_2_ emission level by referring to Xu and Lin et al.^34,35^. The specific measurements are as follows.

2 $${\text{Greenhouse}}\,{\text{gas}}\,\left( {{\text{GHG}}} \right)\,{\text{emissions }} = {\text{ activity}}\,{\text{data }}\left( {{\text{AD}}} \right) \, \times {\text{ emission}}\,{\text{factor }}\left( {{\text{EF}}} \right),$$

where AD is the activity volume of the production or consumption activity that leads to GHG emissions, such as consumption of each fossil fuel, consumption of limestone feedstock, net electricity purchased, net steam purchased, etc (Table 1).

**Table 1:** Carbon emission factor for different types of fuels.

Energy types	Raw coal	Coke	Crude oil	Petrol	Paraffin	Diesel	Fuel oil	Natural gas	Electricity
Converted to standard coal	0.7143	0.9714	1.4286	1.4714	1.4714	1.4571	1.4286	1.33	0.345
Carbon emission factor	0.7559	0.855	0.5857	0.5538	0.5714	0.5921	0.6185	0.4483	0.272

All tables and figures are our creations.

#### Measurement of ICT development level

The level of ICT development (ICT) is the core independent variable of this paper. ICT exists in a variety of forms and has a wide range of applications. Therefore, a composite indicator should be selected to measure the level of ICT development. Referring to the paper by Ding^36^ and Li^37^ and others, this paper selects three dimensions of ICT infrastructure, ICT application level, and ICT service level to construct an evaluation index system. As shown in Table 2, this paper uses the number of Internet broadband accesses as a secondary indicator to measure the level of ICT infrastructure, and selects the number of cell phone owners to measure the level of ICT application, and uses the number of information industry employees to measure the level of ICT services.

**Table 2:** Information and communication technology development level measurement indicators.

Total index	Tier 1 indicators	Indicator calculation
ICT development level	ICT infrastructure level	The number of access users of Internet broadband in the city
	ICT application level	Number of cell phone subscribers in the city at the end of the year
	ICT service level	Number of employees in the information transmission computer services and software industry

Because the measurement results of the entropy value method are more objective and reasonable, it is widely used in the decision analysis of multi-attribute and multi-dimensional indicators. Based on this, this paper adopts the method to calculate the ICT development level.

The entropy method is an objective method of empowerment. It refers to the determination of indicator weights based on the amount of information provided by the observations of each indicator. In its specific use, the entropy method is based on the degree of dispersion of each indicator. The information entropy is used to calculate the weights of the indicators. The weights of each indicator derived from this method are more objective. An important prerequisite for the use of the entropy method is the standardization of the data. An important prerequisite for the use of the entropy method is the standardization of the data. The details are as follows.

$$\mathrm{For \, positive \, indicators{:}}\,Zij=\frac{xij-\mathrm{min}(xj)}{\mathrm{max}\left(xj\right)-\mathrm{min}(xj)},$$

$$\mathrm{For \, negative \, indicators{:}}\, Zij=\frac{\mathrm{max}\left(xj\right)-xij}{\mathrm{max}\left(xj\right)-\mathrm{min}(xj)},$$

#### Control variables

Referring to the current literature, the following control variables were selected to improve the precision of the regression results: (1) Human capital (hca) is represented by the number of students enrolled in higher education in each region. The increase in the level of human capital promotes regional technological innovation, improves the efficiency of factor resource utilization, and reduces energy consumption, which in turn affects urban CO_2_ emissions. (2) Foreign direct investment (fdi) is represented by actual foreign investment used in the year. Foreign direct investment can promote technological innovation through knowledge and technology spillover effects, which is conducive to enhancing urban economic efficiency. (3) Fiscal expenditures (fex) are represented by local government public finance expenditures. Fiscal expenditure growth has a significant contribution to economic growth and regional environmental quality. This suggests that higher fiscal spending may have a positive effect on reducing urban CO_2_ emissions. (4) The level of economic development (gdp), expressed as the city's annual gross product. Shao^38^ argues that GDP can directly reflect the degree of economic development of a region, and the level of economic development directly determines the fiscal revenue of local governments, which affects the investment of environmental protection funds and thus the regional CO_2_ emissions. (5) The well-being of residents (hos) index is represented by the number of hospital beds per capita. The higher the happiness index of residents' life, the more they will develop a certain awareness of environmental protection, which will have a corresponding impact on regional CO_2_ emissions. (6) Environmental regulation (ere) is represented by the municipal wastewater treatment rate. On the one hand, environmental regulation can crowd out innovation input capital, hinder innovation of innovation input capital, and be detrimental to urban environmental protection, while on the other hand, it can help reduce CO_2_ emissions in urban areas by forcing enterprises to innovate in technology and promoting industrial structure transformation and upgrading.

### Data sources

Given the large number of data in the sample of prefecture-level cities selected for this study, certain areas, such as Tibet, have serious data deficiencies. Such cities were therefore excluded and a sample of 269 cities was finally obtained after a series of matching and screening. This paper takes 269 prefecture-level cities in mainland China from 2010 to 2020 to examine the spatial spillover effect of ICT development on regional CO_2_ emissions, with data from 2011 to 2021 China Urban Statistical Yearbook, China Statistical Yearbook, provincial statistical yearbooks of all years, and the China Economic and Social Development Statistical Database, with some missing data to be supplemented by interpolation. The results of the descriptive statistics of the variables are shown in Table 3.

**Table 3:** Results of descriptive statistics of variables.

Variables	Average value	Standard deviation	Minimum value	Maximum value
CO_2_	3.1782	0.7218	1.0186	5.4412
*ICT*	0.1026	0.0929	0.0129	0.9829
*pat*	7.3272	1.6256	2.1972	12.3123
*ind*	1.0448	1.0008	0.1087	36.7921
*hca*	10.6123	1.3543	3.9120	13.9553
*fdi*	0.0036	0.1237	0.0003	0.3656
*fex*	0.1917	0.1077	0.0438	3.4422
*gdp*	10.7010	0.5753	8.8808	13.0557
*hos*	9.6886	0.6975	7.3550	12.0862
*ere*	8.1913	1.1541	1.9459	11.5048

## Empirical results and discussion

### Spatial correlation identification

Based on the previous model setting and testing ideas, this paper refers to Shao Shuai's article^39^ to identify the spatial correlation of variables. And the full domain spatial correlation index is usually measured using Moran's index, as in Eq. (3):

3 $$I = [n\sum\limits_{i = 1}^{n} {\sum\limits_{j = 1}^{n} {W_{ij} (x_{i} - \overline{x} )(x_{j} - \overline{x} } } )]/[\sum\limits_{i = 1}^{n} {\sum\limits_{j = 1}^{n} {W_{ij} } } (x_{i} - \overline{x} )^{2} ].$$

In the formula, n represents the prefecture-level city, *Wij* is the spatial weight, *x* and $\overline{x}$ represents the regional CO_2_ emissions and their regional mean values.

**Figure 2:**
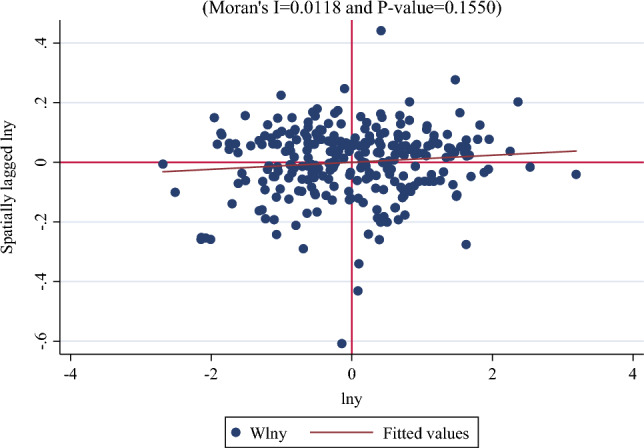
Moran Index.

The identification results of the spatial correlation of Morans’I are shown in Table 4. From 2010 to 2020, all Morans’I results passed the significance test, suggesting that regional carbon emissions are all correlated over spatial distances in all cases; therefore, it is more accurate to use a spatial econometric model to verify the relationship between ICT and regional CO_2_ emissions (Fig. 2).

**Table 4:** Morans’I.

Name	CO_2_		
Time	Moran’I	sd(I)	p-value*
2010	0.012	0.007	0.036
2011	0.012	0.007	0.031
2012	0.012	0.007	0.032
2013	0.011	0.007	0.042
2014	0.012	0.007	0.034
2015	0.012	0.007	0.035
2016	0.012	0.007	0.033
2017	0.012	0.007	0.033
2018	0.012	0.007	0.034
2019	0.012	0.007	0.036
2020	0.011	0.007	0.041

The global spatiality reflects the correlation of spatial variables in the overall space. It is possible to ignore the atypical features of local regions. For the above reasons, the local spatial correlation analysis of CO_2_ emissions is also needed. The most commonly used index is the local Moran's I. In this paper, we refer to Zheng Hao's practice^40^ and establish the following model, as in Eq. (4).

4 $$I_{{\text{i}}} = [(x_{i} - \overline{x} )/S^{2} ] \cdot \sum {_{j \ne i} } W_{ij} (x_{j} - \overline{x} )$$

In the formula,$S^{2} = \sum\nolimits_{i} {[(x_{i} - x)^{2} ]/n}$.

**Figure 3:**
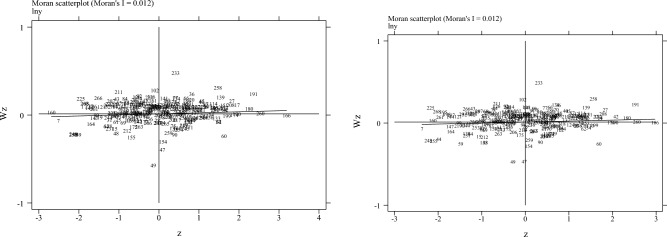
Moran scatter plot for regional CO_2_emission.

In this paper, we examined the characteristics of CO_2_ emissions using the local Moran's I, and the results are shown in Fig. 3. Figure 3 shows that most of the regions are in the first and third quadrants, and more than 50% of the cities have positive Moran'I, indicating that China's current urban CO_2_ emissions are of the high-high or low-low type.

### Results of spatial econometrics analysis

#### Baseline regression result

From the estimated results in Table 4 and Figs. 1 and 2, there may be a significant spatial correlation between ICT development and regional CO_2_ emissions, and thus the results estimated by the traditional OLS model may not be accurate. To make the regression results more accurate, this paper will choose the spatial panel SDM that can comprehensively consider the spatial correlation of economic activities in each city to estimate, and use the spatial fixed effects model. The regression estimation results are shown in Table 5.

**Table 5:** SDM spatial econometric regression results.

Variables	(1)	(2)
ICT	− 0.6072*** (− 6.05)	− 0.7131*** (− 7.18)
hca		0.0242*** (3.96)
fdi		0.0333 (0.17)
fex		− 0.0215 (− 0.69)
gdp		0.0826*** (5.33)
hos		0.0831*** (3.78)
ere		0.0226*** (4.61)
W × ICT	1.2215*** (3.71)	1.0891*** (2.80)
W × hca		− 0.1185 (− 1.64)
W × fdi		− 3.9956** (− 2.25)
W × fex		− 0.0540 (− 0.20)
W × gdp		0.0405 (0.60)
W × hos		− 0.1124* (− 1.76)
W × ere		− 0.0331 (− 1.40)
Control variables	No	Yes
*ρ*	0.7772*** (20.34)	0.4904*** (6.36)
*N*	2959	2959
*R*^2^	0.35	0.23

Standard errors in parentheses *p < 0.1, **p < 0.05, ***p < 0.01.

The regression results of the SDM model are reported in Table 5. The regression results show that ICT development can reduce CO_2_ emissions in the region and increase CO_2_ emissions in neighboring regions. And the results in Table 2 show that the model spatial autoregressive coefficient (*ρ*) is significantly positive, which indicates that the model has not only exogenous interaction effects of the explanatory variables but also endogenous interaction effects of the explained variables. That is, regional CO_2_ emissions themselves have certain spillover effects. Hypothesis 1 in this paper holds.

#### Regression results of other spatial econometric models

The estimation results of the SAR, SEM and SAC models with spatial fixed effects are also presented in this paper, as shown in Table 6. The results show that the coefficients of each spatial econometric model, such as SAR, SEM and SAC, are significantly negative, indicating that ICT development in the region decreases regional CO_2_ emissions when influenced by social and environmental factors in the neighboring regions. The SDM model has the highest number of significant regression coefficients in terms of model fit.

**Table 6:** SAR&SEM&SAC spatial econometric regression results.

	SAR	SEM	SAC
	(1)	(2)	(3)
*ICT*	− 0.6329*** (− 6.57)	− 0.7091*** (− 7.14)	− 0.7086*** (− 7.23)
*ρ*	0.6153***(11.89)		− 1.0753*** (− 5.37)
*λ*		0.6905*** (13.18)	0.9107*** (39.88)
Control variables	Yes	Yes	Yes
*N*	2959	2959	2959
*R*^2^	0.17	0.30	0.28

#### Analysis of direct effects, indirect effects and total effects for SDM

Although the coefficient of the level of ICT development is significantly negative in the spatial Durbin model, and the coefficient of its spatial lag term is significantly positive, this does not explain the spillover effect of ICT development on regional CO_2_ emissions. LeSage and Pace proposed using direct effects to explain the effect of the independent variable in one region on the dependent variable in that region and indirect effects to explain the effect of the independent variable in one region on the dependent variable in other regions, which would avoid drawing wrong conclusions in the analysis. Reference the study of LeSage and Pace^32^, this paper further analyzes the impact of the ICT development level on regional CO_2_ emissions through direct effects, indirect effects and total effects. The regression estimation results are shown in Table 7.

**Table 7:** Results of direct and indirect effects.

Variables	Direct effect		Indirect effects		Total effect	
	Coefficient	Z-value	Coefficient	Z-value	Coefficient	Z-value
*ICT*	− 0.704***	− 6.94	1.421*	1.89	0.717	0.96
*hca*	0.0232***	3.90	− 0.195	− 1.40	− 0.172	− 1.23
*fdi*	0.0238	0.13	− 7.677**	− 2.21	− 7.654**	− 2.20
*fex*	− 0.0225	− 0.74	− 0.149	− 0.28	0.171	− 0.33
*gdp*	0.0832***	5.55	0.161	1.25	0.244*	1.91
*hos*	0.0837***	3.86	− 0.149	− 1.26	− 0.652	− 0.55
*ere*	0.0225***	4.41	− 0.0407	− 0.88	− 0.0182	− 0.39

Firstly, this paper analyzes the impact of ICT development on regional CO_2_ emissions. The results in Table 6 show that ICT development can increase regional CO_2_ emissions in the region, indicating that ICT can reduce regional CO_2_ emissions in the region through technological innovation effects. The spatial spillover effect of ICT is significantly positive, indicating that the development of ICT in this region will increase the regional CO_2_ emissions in neighboring regions. The possible reasons are: The rapid development of information and communication technology and industry will bring a series of technological innovations. And the innovation and upgrading of technology within the industry and enterprises will lead them to a low carbon path, which will lead to a reduction in regional CO_2_ emissions. At the same time, the development of ICT requires a certain amount of capital and manpower investment, which will attract resources from neighboring regions, thus leading to a poorer level of resource allocation and increased levels of CO_2_ emissions in neighboring regions. Next, the parameter estimates of the control variables are analyzed. (1) The direct effect of human capital quality (hca) is significantly positive and the indirect effect is negative, indicating that an increase in human capital quality in cities increases CO_2_ emissions in the region and has a non-significant effect on neighboring regions. (2) The direct effect of foreign direct investment (fdi) is positive and the indirect effect is significantly negative, indicating that the effect of foreign investment on CO_2_ emissions in the region is insignificant, while the effect on carbon emission reduction in neighboring regions is significant. (3) Both the direct and indirect effects of fiscal expenditure (fex) are negative, implying that government fiscal expenditure is beneficial to the reduction of carbon emissions in both the region and the neighboring regions. (4) The direct and indirect effects of economic development (gdp) are both positive, indicating that urban economic development is mostly at the expense of the environment and that the increase in the level of urban economic development is not conducive to the reduction of regional carbon emissions. (5) The direct effect of the happiness index (hos) and environmental regulation (ere) is significantly positive, but the indirect effect does not pass the significance test, indicating that the increase in the happiness index and urban sewage discharge significantly affects the CO_2_ emissions in the region, but does not have a significant spillover to the neighboring areas.

#### Analysis of the mechanism of action

The above paper confirms that ICT development has a significant spatial spillover effect on regional CO_2_ emissions, but what indirect transmission mechanism exists between the two is the key question that needs to be studied next in this paper.

Based on the mechanism analysis in the hypothesis section of the study above, the mediating variables selected in this paper are technological innovation (pat) and industrial upgrading (ind) respectively. Technological innovation is represented by the number of annual patents granted in the city, following Wang^41^, while industrial upgrading is represented by the ratio of value added in the tertiary sector to value added in the secondary sector, following Zhang^42^. Meanwhile, to reduce the impact of heteroskedasticity on the data, the data are all logarithmic in this paper. Therefore, to verify whether there is a mediating effect of technological innovation and industrial upgrading in the mechanism of ICT influence on regional CO_2_ emissions, the model in this paper is established as follows.

5 $$lnY_{it} = \alpha_{0} + \delta_{1} WlnY_{it} + \alpha_{1} lnX_{it} + \alpha_{2} lnX_{Control} + \alpha_{3} WlnX_{it} + \alpha_{4} WlnX_{control} + \varepsilon_{it} ,$$

6 $$lnM_{it} = \beta_{0} + \delta_{2} WlnM_{it} + \beta_{1} lnX_{it} + \beta_{2} lnX_{Control} + \beta_{3} WlnX_{it} + \beta_{4} WlnX_{control} + \varepsilon_{it} ,$$

7 $$lnY_{it} = \, c_{0} + \delta_{3} WlnY_{it} + c_{1} lnX_{it} + c_{2} lnX_{Control} + c_{3} WlnX_{it} + c_{4} WlnX_{control} + c_{5} lnM_{it} + c_{6} WlnMit + \varepsilon_{it} .$$

The results of this paper are shown in Table 8, which shows that the level of ICT development has a significant positive effect on technological innovation and industrial upgrading. This result satisfies the basic conditions for the mediating effect model to be valid. At the same time, the regression coefficients γ are all significant, which means that there is a mediating transmission mechanism. The above results indicate that technological innovation and industrial upgrading have a mediating effect in the mediating transmission mechanism between the ICT development level and regional CO_2_ emissions. The level of ICT development in the region can increase the CO_2_ emissions in neighboring regions through technological innovation and industrial upgrading. The possible reasons for this are as follows. The increase in the level of technology in the region and the change in industrial structure can lead to high-tech and low-carbon enterprises moving into the region, while neighboring regions have more traditional and highly polluting enterprises, thus increasing CO_2_ emissions.

**Table 8:** Analysis of the mechanism of action.

	pat		ind	
	(1)	(2)	(1)	(2)
*ICT*	4.6997*** (12.59)	− 0.4750*** (− 4.75)	0.8124* (1.81)	− 0.7119*** (− 7.17)
*M*		− 1.0494*** (− 10.29)		0.0065 (1.59)
*W* × *ICT*	− 7.2763*** (− 4.83)	1.3141*** (3.13)	− 5.2299*** (− 2.85)	1.6276*** (3.66)
*W* × *M*		0.1361*** (3.13)		0.0614* (2.27)
*ρ*	0.5167*** (6.22)	0.4541*** (5.75)	0.3569*** (4.13)	0.3986*** (4.48)
Control variables	Yes	Yes	Yes	Yes
*N*	2959	2959	2959	2959
*R*^*2*^	0.42	0.23	0.45	0.24

### Robustness tests

#### Change the spatial weight matrix

The previous study constructed the spatial weight matrix in terms of spatial geographical distances and did not consider the spatial correlation arising from actual geographical distances. In this paper, we refer to the study of Shao et al.^38^ to construct an actual distance weight matrix, which better fits the actual distance situation, as a way to test whether the results are stable. Verify again that the conclusions are still robust. The empirical results are shown in Table 9. After using the geographic distance weight matrix, the regression results are generally consistent with the above. This also indicates that the results of the study are robust and reliable.

**Table 9:** Economic distance matrix regression results.

	(1)	(2)
ICT	− 0.6003*** (− 6.01)	− 0.7035*** (− 7.11)
W × ICT	1.0478*** (3.02)	0.9960** (2.21)
*ρ*	0.8443*** (25.13)	0.4784*** (4.13)
Control variables	No	Yes
*N*	2959	2959
*R*^*2*^	0.36	0.24

#### Shortened period

To further ensure the reliability of the empirical results of the article, the sample intervals are changed in this paper to conduct the robustness analysis. That is, by delineating different sample time intervals to ascertain that the results of the baseline regression are not generated from a specific sample interval. Specifically, only the sample from 2011 to 2017 was retained for the regressions and the results are shown in Table 10. It can be found that after changing the sample interval, the positive and negative regression coefficients are consistent with those above, indicating that the results of the benchmark regression are convincing.

**Table 10:** Shortened period regression results.

	(1)	(2)
*ICT*	− 0.3318*** (− 4.82)	− 0.3664*** (5.33)
*W* × *ICT*	1.0402*** (3.96)	1.4975*** (3.99)
*ρ*	0.6555*** (9.32)	0.3975*** (3.50)
Control variables	No	Yes
*N*	1883	1883
*R*^*2*^	0.33	0.22

### Heterogeneity analysis

#### Regional heterogeneity analysis

1. East-Central-West Heterogeneity

China has a long history of uneven development between regions, resulting in varying levels of ICT development between the eastern and central-western regions. The level of regional CO_2_ emissions also varies greatly from region to region. This section attempts to conduct a regression analysis of regional heterogeneity in the impact of ICT on regional CO_2_ emissions. The results of the correlation measure regressions are shown in columns (1) and (2) of Table 11. From the regression results, it can be concluded that the level of ICT development in the eastern region increases CO_2_ emissions in the region and neighboring regions. The level of ICT development in western cities will reduce CO_2_ emissions in the region and increase CO_2_ emissions in neighboring areas. Possible reasons for this are listed below. The cities in the eastern region are ripe for ICT development and have developed to saturation. The laying of too much information infrastructure and the use of information equipment can lead to a rapid increase in energy consumption in the ICT sector, thus increasing the regional carbon footprint of the region and also having a bad impact on neighboring areas. The western cities, on the other hand, are at a stage of development where ICT is taking advantage of its convenience and effect to speed up economic exchanges and thus reduce the region's carbon footprint.

(B) North–South heterogeneity

**Table 11:** Regional heterogeneity test.

Variables	(1)	(2)	(3)	(4)
	Eastern Cities	Midwestern Cities	Southern Cities	Northern Cities
*ICT*	0.4576*** (5.78)	− 0.5276*** (− 4.84)	0.2807*** (3.16)	0.3668*** (4.24)
*W* × *ICT*	0.9229*** (3.31)	1.3786*** (3.86)	− 1.2195*** (− 6.38)	2.2841*** (5.79)
*ρ*	− 0.4414** (− 2.14)	0.3143*** (3.38)	− 0.0017 (− 0.01)	− 0.6544*** (− 3.63)
Control variables	Yes	Yes	Yes	Yes
*N*	1298	1661	1144	1815
*R*^*2*^	0.51	0.04	0.57	0.29

The difference between the north and south is the most important regional difference in China. The Qinling Mountains-Huaihe River Line is the main boundary dividing the country of China into north and south. There are significant differences between the natural and human landscapes north of the Qinling-HuaiheLine and south of the Qinling-HuaiheLine. This section attempts a regression analysis of the north–south variability in regional CO_2_ emissions as influenced by ICT. The results of the correlation measure regressions are shown in columns (3) and (4) of Table 11. The regression results show that the level of ICT development in the southern cities significantly increases CO_2_ emissions in the region and reduces CO_2_ emissions in the neighboring areas. In contrast, the level of ICT development in the northern cities significantly increases CO_2_ emissions in neighboring areas, with no significant impact on the region. Possible reasons are as follows. The southern region is mostly a coastal city in China with a high level of urban economic development. Too many companies located in the southern region will increase the environmental burden in the region. In turn, government departments in neighboring regions will introduce policies to force the development of new technologies and focus on reducing regional CO_2_ emissions. Most of the northern cities are inland cities with more backward economic development. Government departments focus on the speed of economic development at the expense of the environment.

#### Environmental information disclosure heterogeneity analysis

The effects of environmental regulation often vary depending on the institutional basis. In 2008, the State promulgated the Measures on Environmental Information Disclosure, which requires 113 cities nationwide to disclose environmental information regularly. Transparency of environmental information can enhance the motivation of local officials for environmental governance and establish long-term environmental governance mechanisms (Anderson et al. 2019). This paper next develops a heterogeneity analysis based on whether local environmental information is publicly available. The regression results are shown in columns (1) and (2) of Table 12. The level of ICT development in cities with open environmental information reduces carbon emissions in the region and neighboring areas. In contrast, the level of ICT development in cities with undisclosed environmental information has some dimensions of increasing carbon emissions in the region and neighboring areas. Therefore, in the subsequent process of carbon emission management, the country needs to further strengthen the environmental information disclosure and improve the environmental quality standard system.

**Table 12:** Environmental information disclosure heterogeneity test.

Variables	(1)	(2)
	Environmental information disclosure cities	Undisclosed environmental information cities
*ICT*	− 0.0771*** (− 3.36)	0.0117 (1.36)
*W* × *ICT*	− 1.8103*** (− 4.39)	0.1054 (0.56)
*ρ*	− 0.9171** (− 2.46)	− 0.6059* (− 1.88)
Control variables	Yes	Yes
*N*	1210	1749
*R*^*2*^	0.09	0.04

### Discussion

#### According to the above research, we find some interesting phenomena

First, although the development of ICT is beneficial to carbon reduction in the region, it has significant spillover effects that undermine carbon reduction in neighboring regions. This view is at variance with what scholars have thought in the past. We usually think that the development of ICT is beneficial to accomplish carbon reduction. This result triggers us to think about the development of technological progress. The rapid development of technology has facilitated people's lives but is it at the expense of our environment? A deeper thought is needed.

Second, the heterogeneity results in this paper indicate that the carbon reduction effect is more pronounced in cities with publicly available environmental information, while there is no carbon reduction effect in cities without public disclosure. This finding is consistent with existing theories of environmental regulation and policy intervention. It shows that reasonable government intervention is very effective in the process of economic and social development. The government needs to adjust its approach promptly in response to various problems in economic development.

## Conclusions and policy recommendations

This paper studies the spatial spillover effect of urban ICT development level on regional CO_2_ emissions by using a spatial econometric model with panel data from 269 cities in China from 2010 to 2020 as a sample. The article also measures the direct, indirect and total effects of each factor on regional CO_2_ emissions, and enhances the credibility of the conclusions through a series of robustness tests. It also extends the analysis from a heterogeneity perspective. The main conclusions of this paper are as follows.

First, the spatial data analysis shows that the CO_2_ emissions of Chinese cities are all correlated in spatial distance and show high-high type or low-low type clustering characteristics.

Second, the level of ICT development can significantly reduce the regional CO_2_ emissions in the region, while it has a significant positive spillover effect on the urban CO_2_ emissions in neighboring regions. And the results still hold after passing the robustness test.

Third, this paper further explores the mechanism of the interaction between ICT development level and regional CO_2_ emissions and finds that ICT can influence regional CO_2_ emissions through its technological innovation effect and industrial upgrading effect.

Fourth, the results of regional heterogeneity analysis show that the level of ICT development in the eastern region increases CO_2_ emissions in the region and neighboring regions, while the level of ICT development in the western region decreases CO_2_ emissions in the region and increases CO_2_ emissions in neighboring regions.

Fifth, the results of environmental information disclosure heterogeneity show that the level of ICT development in cities where environmental information is disclosed reduces carbon emissions in the region and neighboring areas. And the level of ICT development in cities with undisclosed environmental information increases carbon emissions in the region and neighboring areas.

Based on the above empirical results, this paper puts forward the following policy recommendations from the perspective of the whole country and the four major regions:

First, government departments and enterprises should pay attention to the energy consumption and impact on greenhouse gases brought by the ICT industry itself, and encourage ICT industries to establish long-term renewable energy development strategies. At the same time, cities should actively study the potential of applying ICT to reduce CO_2_ emissions in various industries so that reasonable policies can be formulated to guide the development and promote the arrival of a low-CO_2_ economy.

Second, the government should pay attention to the technological innovation effect and industrial upgrading effect of the ICT industry. It is necessary to improve the employment environment for researchers and motivate them to conduct research as a way to promote high-quality development. The government also needs to focus on the development of high-tech, low-carbon enterprises and adopt environmental regulations to force the transformation of more polluting enterprises. We play a driving role in environmental regulation to reduce regional carbon emissions and establish a long-term environmental governance pattern in line with Chinese characteristics.

Third, the impact of ICT development level on regional CO_2_ emissions has obvious heterogeneous characteristics. Regions rich in renewable energy should consider prioritizing the use of renewable energy when formulating energy-saving policies for ICT industries, making full use of the agglomeration effect of resource-based cities on their advantageous economic resources to promote their CO_2_ emission reduction while spreading to their neighboring cities, which not only relieves their environmental pressure but also drives the environmental management of neighboring cities.

Finally, the establishment of a regional joint prevention and control mechanism for CO_2_ emission reduction to form a regional synergy for effective CO_2_ reduction. The spatial spillover effects of regional CO_2_ emissions dictate that "unilateral" CO_2_ reduction efforts may be futile due to the "leakage effect" of CO_2_ emissions between regions. Therefore, the CO_2_ emission reduction policy must be based on the premise of regional joint prevention and control to be effectively implemented. We should increase regional joint environmental law enforcement and supervision, establish a unified monitoring platform for environmental pollution, implement regional environmental information sharing mechanism, joint early warning mechanism, demonstration effect mechanism, and so on. Only by forming regional synergies can we contribute to CO_2_ reduction.

## Data Availability

The data selected in this paper are all from China City Statistical Yearbook, China Statistical Yearbook, China Environmental Yearbook and China Statistical Yearbook by Province repository. The data that support the findings of this study are available from (https://data.cnki.net/). All data analyzed during this study are included in this published article and its supplementary information files. The datasets used and analyzed during the current study are available from the corresponding author upon reasonable request.
